# Global prevalence of needlestick and sharps injuries among dental health care workers: A systematic review and meta-analysis

**DOI:** 10.1097/MD.0000000000051365

**Published:** 2026-10-30

**Authors:** Xiaodan Zhu, Weili Ding, Mao Ye, Ling Zhang, Bei Zhang, Min Xing, Wenhao Qian

**Affiliations:** a Department of Nursing, Laboratory of Dental Biomaterials and Tissue Regeneration, Shanghai Xuhui District Stomatological Hospital, Shanghai, P.R. China; b Department of Endodontics, Laboratory of Dental Biomaterials and Tissue Regeneration, Shanghai Xuhui District Stomatological Hospital, Shanghai, P.R. China; c Department of Science and Education, Laboratory of Dental Biomaterials and Tissue Regeneration, Shanghai Xuhui District Stomatological Hospital, Shanghai, P.R. China; d Office of the President, Laboratory of Dental Biomaterials and Tissue Regeneration, Shanghai Xuhui District Stomatological Hospital, Shanghai, P.R. China.

**Keywords:** dental health care workers, meta-analysis, needlestick and sharps injuries, systematic review

## Abstract

**Background:**

To explore the prevalence of needlestick and sharps injuries (NSI) among dental health care workers (DHCWs) across different countries and regions worldwide.

**Methods:**

Observational studies reporting NSI prevalence among DHCWs were included. A meta-analysis was conducted based on data retrieved from 6 databases (PubMed, Web of Science, Embase, Medline-Ovid, Cumulative Index to Nursing and Allied Health Literature, and Cochrane Library). The study was registered in International Prospective Register of Systematic Reviews (CRD42024598365). The quality of included studies was evaluated using the Joanna Briggs Institute Critical Appraisal Checklist for Studies Reporting Prevalence Data. A random-effects model was used to estimate the pooled prevalence. Publication bias was assessed using a funnel plot and Begg’s test. Sensitivity analysis was performed using the leave-one-out approach and the trim and fill method. Searches were conducted through October 13, 2024.

**Results:**

A total of 56 studies comprising 21,481 participants from 27 countries and regions were included, with the reported prevalence ranging from 11.59% (Australia) to 89.17% (Greece). The pooled prevalence of NSI was 44.11% (95% confidence interval [CI]: 38.01–50.30%) among DHCWs, with significant heterogeneity (*I*^2^ = 98.77%, *P* < .001). Subgroup analyses yielded the following pooled prevalences: dentists 49.41% (95% CI: 38.81–60.03%), dental students 43.12% (95% CI: 36.91–49.45%), nurses 40.85% (95% CI: 21.48–61.80%), and other DHCWs 33.75% (95% CI: 14.21–56.73%). This review had substantial heterogeneity (*I*^2^ = 98.77%) and was limited by the observational design of included studies.

**Conclusion:**

The prevalence of NSI among DHCWs is relatively high. This issue warrants urgent attention. Safety-engineered devices are recommended as the primary engineering control. Hospital management should establish and improve NSI reporting systems. Training and competency assessment are necessary for all DHCWs. This work was supported by Xuhui District Dental Disease Prevention and Treatment Institute, Shanghai (SHXYF202312).

## 1. Introduction

Needlestick and sharps injuries (NSI) refer to accidental events in which healthcare workers are punctured or cut by sharp medical instruments during their work. Such injuries increase the risk of infection by pathogenic microorganisms. A meta-analysis on the global prevalence of needlestick injuries included 113 studies and 5,25,798 healthcare workers, indicating that the prevalence of NSI among healthcare workers is as high as 43%.^[1]^ Further research by Bouya et al found that the prevalence of NSI among dental health care workers (DHCWs) was the highest (59.1%) among all clinical departments.^[2]^ However, these data are derived solely from annual surveillance reports of needlestick injuries. Some studies have shown that more than 20% of individuals fail to report or deliberately conceal these incidents; therefore, the actual prevalence of NSI may be substantially higher.^[3,4]^

NSI poses a serious threat to the physical and mental health of healthcare workers. Such injuries represent one of the primary routes through which healthcare workers become infected with bloodborne pathogens (BBPs). This risk is further increased by the high prevalence of human immunodeficiency virus (HIV), hepatitis B virus (HBV), and hepatitis C virus (HCV) among outpatients.^[5]^ Moreover, patients carrying HBV, HCV, and HIV frequently conceal their infection status when seeking treatment.^[6]^ In occupational exposures resulting from sharps injuries, HBV is the predominant BBP, accounting for an exposure rate of 54.9%, and HIV ranks second with 12.3%.^[7]^ In addition, NSI imposes substantial social and economic burdens. The economic burden of NSI in countries such as China and Japan is estimated at hundreds of millions of dollars per year.^[8,9]^

This meta-analysis synthesizes evidence from multiple studies to estimate NSI prevalence among dental personnel across countries and regions and to explore differences by occupation and setting. These findings may inform targeted vocational training and prevention strategies to reduce the NSI burden in dental settings.

## 2. Materials and methods

### 2.1. Registration and protocol

This systematic review and meta-analysis was registered in the International Prospective Register of Systematic Reviews (CRD42024598365). This study is based on published literature; therefore, ethical approval was not required. The review protocol is available from the corresponding author upon reasonable request.

### 2.2. Eligibility criteria

Inclusion criteria: original articles published from the inception of each database to October 13, 2024, with full text available. Observational studies (cross-sectional, descriptive, prospective, cohort, and case study designs) were eligible. Studies were required to report the prevalence of NSI among DHCWs (including dentists, dental students [DSS], dental nurses, and dental assistants).

Exclusion criteria: case-control studies, randomized controlled trials, interventional studies, commentaries, expert opinions, theses, editorials, book chapters, letters to the editor, brief reports, and reviews.

### 2.3. Search strategy

The search strategy was: (needlestick injuries OR percu* injur* OR percutaneous exposure* OR occupation* exposure OR accident* exposure OR accidental occupational OR exposure needle injur* OR needlestick injur* OR needle stick injur* OR needle-stick injur* OR sharp* injur* OR occupation* injur* OR body fluid* exposure) AND (dental OR dentist OR stomatolog*) AND (rate* OR risk* OR ratio*). The sources were searched across 6 databases (PubMed, Web of Science, Embase, Medline-Ovid, Cumulative Index to Nursing and Allied Health Literature, and Cochrane Library) from the inception of each database to October 13, 2024. In addition, citation searching of included articles was performed to identify potentially relevant studies.

### 2.4. Selection of studies and data extraction

All records were imported into EndNote (version X9) to remove duplicates. Two reviewers independently conducted study screening, eligibility assessment, and data extraction. First, titles and abstracts were screened to assess preliminary eligibility. Next, full texts were reviewed to select relevant literature according to the inclusion and exclusion criteria. Finally, relevant information was extracted from the included studies. Disagreements were resolved by consensus with a third reviewer. The extracted information included: first author, year of publication, country, sample size, response rate, prevalence rate, source of occurrence data, and study population.

### 2.5. Risk of bias (quality) assessment

Two reviewers independently evaluated the quality of the literature based on the Joanna Briggs Institute Critical Appraisal Checklist for Studies Reporting Prevalence Data. The checklist comprises 9 items, each rated as yes, no, unclear, or not applicable:

Q1: Was the sample frame appropriate to address the target population?

Q2: Were study participants sampled in an appropriate way?

Q3: Was the sample size adequate?

Q4: Were the study subjects and the setting described in detail?

Q5: Was the data analysis conducted with sufficient coverage of the identified sample?

Q6: Were valid methods used for the identification of the condition?

Q7: Was the condition measured in a standard, reliable way for all participants?

Q8: Was there appropriate statistical analysis?

Q9: Was the response rate adequate, and if not, was the impact of the low response rate addressed?

### 2.6. Data synthesis

All statistical analyses were conducted using R Statistical Software (v4.3.2; R Core Team 2024), R Foundation for Statistical Computing. Heterogeneity between studies was assessed using the *I*^2^ statistic and Cochran’s *Q* test. Given the substantial heterogeneity of prevalence data across different global populations, a random-effects model (DerSimonian–Laird method) was adopted as the primary analytical approach for all pooled estimates. Prevalence data were transformed using the Freeman–Tukey double arcsine transformation prior to pooling. Subgroup analyses were conducted to examine the influence of study characteristics (occupation and geographical region) on the pooled prevalence. To evaluate publication bias, we used a funnel plot and Begg’s test. Sensitivity analysis was performed using the leave-one-out approach and the trim and fill method to assess the stability of the results.

The certainty of evidence for the pooled prevalence estimate was assessed using the GRADE approach adapted for prevalence reviews. Starting from a baseline rating of “low” (observational study design), the evidence was downgraded by 2 levels for very serious inconsistency (*I*^2^ = 98.77%), resulting in an overall certainty rating of “very low.” Subgroup analyses by occupational group (dentists, DSS, nurses, other DHCWs) partially explained the heterogeneity, but residual between-study variance remained substantial. The evidence was not downgraded further for imprecision (total sample size = 21,481, exceeding the optimal information size threshold), indirectness (all studies directly measured NSI prevalence among DHCWs), or publication bias (symmetrical funnel plot, Begg’s test *P* > .05). No upgrade factors were identified. The final GRADE rating is summarized in Table 1.

**Table 1:** GRADE evidence profile for the pooled prevalence of NSI among DHCWs.

Number of studies	Study design	Risk of bias	Inconsistency	Indirectness	Imprecision	Publication bias	Outcome	Pooled prevalence (95% CI)	Sample size (studies)	Quality
56	Observational	Not serious*	Very serious†	Not serious‡	Not serious§	Undetected∥	NSI among DHCWs	44.11% (38.01–50.30%)	21,481 (56)	⊕⊖⊖⊖ VERY LOW

GRADE Working Group grades: High = ⊕⊕⊕⊕; Moderate = ⊕⊕⊕⊖; Low = ⊕⊕⊖⊖; Very low = ⊕⊖⊖⊖. Starting from “low” (observational study design), downgraded 2 levels for very serious inconsistency (*I*^2^ = 98.77%) → final rating: VERY LOW.

CI = confidence interval, DHCW = dental health care worker, JBI = Joanna Briggs Institute, NSI = needlestick and sharps injuries.

* Mean JBI score 7.7/9; 48/56 (85.7%) scored ≥7/9. Most common limitation: inadequate response rate reporting (Q9).

† Very high heterogeneity (*I*^2^ = 98.77%, *Q* = 4464.19, *P* < 0.001). Subgroup analyses by occupational group partially explained the heterogeneity but residual variance remained substantial.

‡ All studies directly measured NSI prevalence among DHCWs.

§ Total sample size = 21,481; 95% CI (38.01–50.30%) considered acceptable for a prevalence estimate.

∥ Symmetrical funnel plot; Begg’s test showed no significant publication bias (*P* > .05).

## 3. Results

### 3.1. Study selection

A total of 2137 records were identified from 6 databases. After removing duplicates, 1274 records were screened by title and abstract; 1179 were excluded, leaving 95 reports sought for retrieval, of which 7 could not be retrieved. The remaining 88 full-text reports were assessed for eligibility, and 37 were excluded. Additionally, 11 records were identified through citation searching; of these, 9 were sought for retrieval, 2 could not be obtained, and 7 were assessed for eligibility, of which 2 were excluded. Ultimately, 56 studies were included in the meta-analysis (Fig. 1).

**Figure 1.**
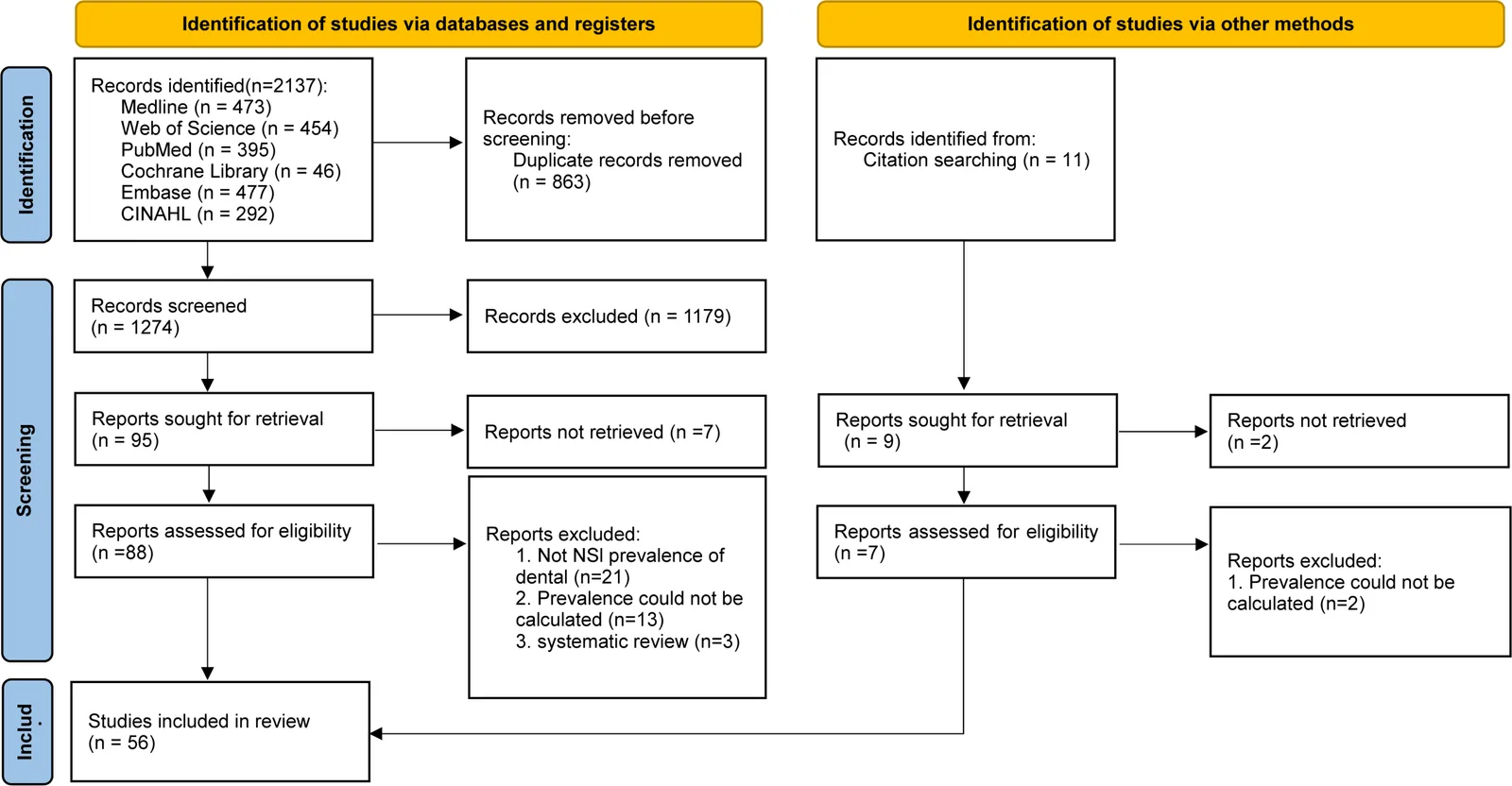
Flow diagram of literature search and screening process. CINAHL = Cumulative Index to Nursing and Allied Health Literature.

### 3.2. Study characteristics

This review included 56 articles, comprising 21,481 DHCWs from 27 countries and regions, with the reported prevalence ranging from 11.59% to 89.17%. The included studies comprised retrospective investigations and questionnaire-based surveys. The basic characteristics and risk of bias assessment results of the included literature are shown in Table 2.

**Table 2:** Summary of descriptive characteristics and bias assessment of included literature.

First author	Year	Country	Sample size	Response rate	NSI prevalence	Source of data	Study population	Q1	Q2	Q3	Q4	Q5	Q6	Q7	Q8	Q9
Gershon	1998	USA	392	60.00%	16.08%	QS	Dentists	+	−	+	+	+	+	+	+	−
McCarthy	1999	Canada	4107	66.00%	61.99%	QS	Dentists	+	−	+	+	+	+	+	+	−
Chowanadisai	2000	Thailand	164	100.00%	50.00%	QS	Dentists	+	+	+	+	+	+	+	+	+
Stewardson	2002	UK	185	100.00%	39.90%	QS	DSS	+	+	+	+	+	+	+	+	+
Veronesi	2004	Italy	122	30.50%	72.14%	QS	Dentists	+	+	+	−	+	+	+	+	−
Al-Sarheed	2004	Saudi Arabia	266	90.90%	68.00%	QS	DSS	+	+	+	+	+	+	+	+	+
Kotelchuck	2004	USA	204	28.00%	32.80%	QS	DSS	−	−	−	+	+	+	+	+	−
Smith	2006	India	107	71.00%	40.02%	QS	DSS	+	+	+	−	+	+	+	+	?
Leggat	2006	Australia	283	73.10%	27.70%	QS	Dentists	+	+	+	+	+	+	+	+	?
Shah	2006	USA	924	NR	20.00%	RS	DHCW	+	+	+	+	−	+	+	+	/
Sofola	2007	Nigeria	183	84.50%	58.80%	QS	DSS	+	+	−	+	+	+	+	+	+
Scully	2007	UK	432	63.50%	34.63%	QS	Dentists	+	+	+	+	+	+	+	+	−
AlNegrish	2008	Jordan	279	93.00%	56.28%	QS	Dentists	+	+	+	+	+	+	+	+	+
Machado	2008	Brazil	286	86.40%	29.00%	QS	DSS	+	+	+	+	+	+	+	+	+
Khader	2009	Jordan	170	94.40%	66.50%	QS	Dentists	+	+	+	+	−	+	+	+	+
Resende	2009	Brazil	1302	93.90%	85.24%	QS	Dentists	+	+	+	+	+	+	+	+	+
Salehi	2010	Afghanistan	676	71.10%	75.40%	QS	Dentists	−	−	−	+	−	+	+	+	?
Mungure	2010	Kenya	72	81.00%	29.00%	QS	DSS	−	−	−	−	+	+	+	+	−
Wicker	2010	Germany	265	58.89%	54.30%	QS	DHCW	+	+	+	+	−	+	+	+	−
Jaber	2011	Saudi Arabia	230	92.00%	23.00%	QS	DSS	+	+	+	+	+	+	+	+	+
Rimkuviene	2011	Lithuania	1446	64.70%	78.50%	QS	Dentists	+	+	+	+	+	+	+	+	−
Gaballah	2012	UK	235	NR	25.50%	RS	DSS	+	+	+	−	+	+	+	+	/
Al-Ali	2012	UAE	733	87.00%	41.75%	QS	Dentists	+	+	+	+	+	+	+	+	+
Askarian	2012	Iran	137	51.90%	73.70%	QS	DSS	+	+	+	+	+	+	+	+	−
Myers	2012	USA	215	72.10%	16.30%	QS	DSS	+	+	+	+	+	+	+	+	+
Al-Dharrab	2012	Saudi Arabia	402	NR	48.50%	QS	Dentists	+	+	+	+	+	+	+	+	?
Cheng	2012	Taiwan, China	434	74.80%	23.00%	QS	Dentists	+	+	+	+	+	+	+	+	?
Sedky	2013	Egypt	350	90.00%	61.90%	RS	DSS	+	+	+	−	+	+	−	−	/
Prabhu	2014	India	102	73.00%	33.40%	QS	Nurses	−	−	?	+	+	+	+	+	?
Zarra	2014	Greece	120	84.00%	89.17%	QS	Dentists	+	+	+	+	+	+	+	+	+
Lee	2014	Taiwan, China	228	NR	21.28%	RS	DHCW	−	−	−	+	+	+	+	+	/
Leavy	2014	UK	293	42.00%	34.13%	QS	Dentists	+	+	+	+	+	+	+	+	−
Kuru	2014	Turkey	556	100.00%	74.60%	QS	DHCW	+	+	+	+	+	+	+	+	+
Shaghaghian	2015	Iran	191	80.00%	43.50%	QS	DSS	+	+	+	+	+	+	+	+	+
Pavithran	2015	India	200	81.50%	27.50%	QS	DHCW	+	+	+	−	+	+	+	+	+
Halboub	2015	Yemen	145	72.00%	62.80%	QS	DSS	+	+	+	+	+	+	+	+	?
Arheiam	2015	Libya	340	100.00%	35.29%	QS	Dentists	+	+	+	+	+	+	+	+	+
Al-Maweri	2015	Saudi Arabia	512	85.00%	34.20%	QS	DSS	+	+	+	+	+	−	−	+	+
Kumar	2016	India	100	NR	35.00%	QS	DSS	?	+	?	+	+	+	+	−	?
Wu	2016	China	171	83.00%	34.00%	QS	DSS	+	+	+	+	+	+	+	−	+
Pinelli	2016	Brazil	173	75.90%	40.00%	QS	DSS	+	+	+	+	+	+	+	+	?
Fernandes	2017	Brazil	137	NR	43.10%	RS	DSS	+	+	+	−	+	+	+	+	/
Al-Essa	2017	Saudi Arabia	363	83.40%	65.00%	QS	DSS	+	+	+	+	+	+	+	+	+
Hbibi	2018	Morocco	83	70.90%	42.70%	QS	DSS	+	+	−	+	+	+	+	+	?
Gilavand	2018	Iran	124	100.00%	45.20%	QS	DSS	+	+	+	+	+	+	+	+	+
Al-Zoughool	2018	Saudi Arabia	515	100.00%	23.00%	QS	Dentists	+	+	+	+	+	+	+	+	?
Madhumitha	2019	India	100	NR	70.00%	QS	DSS	?	+	?	+	+	+	+	+	?
AlDakhil	2019	Saudi Arabia	450	90.00%	29.80%	QS	Nurses	+	+	+	+	+	+	+	+	+
Musekene	2020	South Africa	256	99.00%	41.00%	QS	DSS	+	+	+	+	+	+	+	+	+
Joshi	2021	India	150	71.43%	66.70%	QS	DSS	+	+	+	−	+	+	+	+	?
Yuan	2022	China	257	97.00%	59.92%	QS	Nurses	+	+	+	+	+	+	+	+	+
Huang	2022	China	268	90.00%	36.20%	QS	DSS	+	+	+	+	+	+	+	+	+
Huynh	2022	Australia	345	70.80%	11.59%	RS	DHCW	+	+	+	+	+	+	+	+	/
Wong	2023	Singapore	63	95.45%	15.60%	RS	Dentists	+	+	/	+	+	+	+	+	/
Huang	2023	China	407	91.90%	23.80%	QS	DSS	+	+	+	+	+	+	+	+	+
Thekkiniyakath	2023	Saudi Arabia	231	100.00%	34.63%	QS	DSS	+	+	+	+	+	+	+	+	+

Bias assessment was performed using the JBI Critical Appraisal Checklist for Studies Reporting Prevalence Data (Q1–Q9).

+ = yes, − = no, ? = unclear, / = not applicable, DHCW = dental health care worker, DSS = dental students, JBI = Joanna Briggs Institute, NR = not reported, QS = questionnaire survey, RS = retrospective study.

### 3.3. Prevalence of NSI

The overall prevalence of NSI was 44.11% (95% confidence interval [CI]: 38.01–50.30%), with significant heterogeneity (*I*^2^ = 98.77%, *Q* = 4464.19, *P* < .001), as shown in Figure 2. The country-specific pooled prevalence of NSI is shown in Table 3 and Figure 3. Greece reported the highest prevalence of 89.17% (95% CI: 83.6–94.7%; Table 3 and Fig. 3). Subgroup analysis was conducted among different dental occupational groups (Fig. 4). The pooled prevalence was 49.41% (95% CI: 38.81–60.03%) among dentists (19 studies, *I*^2^ = 99.2%). The pooled prevalence was 43.12% (95% CI: 36.91–49.45%) among DSS (28 studies, *I*^2^ = 95.8%). The pooled prevalence was 40.85% (95% CI: 21.48–61.80%) among nurses (3 studies, *I*^2^ = 96.9%). The pooled prevalence was 33.75% (95% CI: 14.21–56.73%) among other DHCWs (6 studies, *I*^2^ = 99.2%; Fig. 4).

**Table 3:** Country-specific pooled prevalence of NSI.

Country	Number of articles	Pooled prevalence (95% CI)	Country	Number of articles	Pooled prevalence (95% CI)	Country	Number of articles	Pooled prevalence (95% CI)
Yemen	1	62.8% (54.9%, 70.7%)	Turkey	1	74.6% (71.0%, 78.2%)	Taiwan, China	2	22.4% (19.2%, 25.6%)
Afghanistan	1	75.4% (72.2%, 78.6%)	Iran	3	54.2% (34.4%, 74.0%)	Nigeria	1	58.8% (51.7%, 65.9%)
Brazil	4	49.4% (15.5%, 83.3%)	Italy	1	72.1% (64.2%, 80.1%)	Saudi Arabia	8	41.6% (30.8%, 52.3%)
Canada	1	62.0% (60.5%, 63.5%)	Jordan	2	61.2% (51.2%, 71.2%)	UK	4	33.3% (28.0%, 38.7%)
China	4	38.4% (22.7%, 54.2%)	Kenya	1	29.0% (18.5%, 39.5%)	South Africa	1	41.0% (35.0%, 47.0%)
Germany	1	54.3% (48.3%, 60.3%)	Libya	1	35.3% (30.2%, 40.4%)	Thailand	1	50.0% (42.3%, 57.7%)
Greece	1	89.2% (83.6%, 94.7%)	Lithuania	1	78.5% (76.4%, 80.6%)	USA	4	20.8% (15.3%, 26.3%)
India	6	45.7% (30.5%, 61.0%)	Morocco	1	42.7% (32.1%, 53.3%)	UAE	1	41.7% (39.2%, 44.3%)
Egypt	1	61.9% (56.8%, 67.0%)	Australia	2	18.9% (6.0%, 36.7%)	Singapore	1	15.6% (6.6%, 24.6%)

CI = confidence interval, NSI = needlestick and sharps injuries.

**Figure 2.**
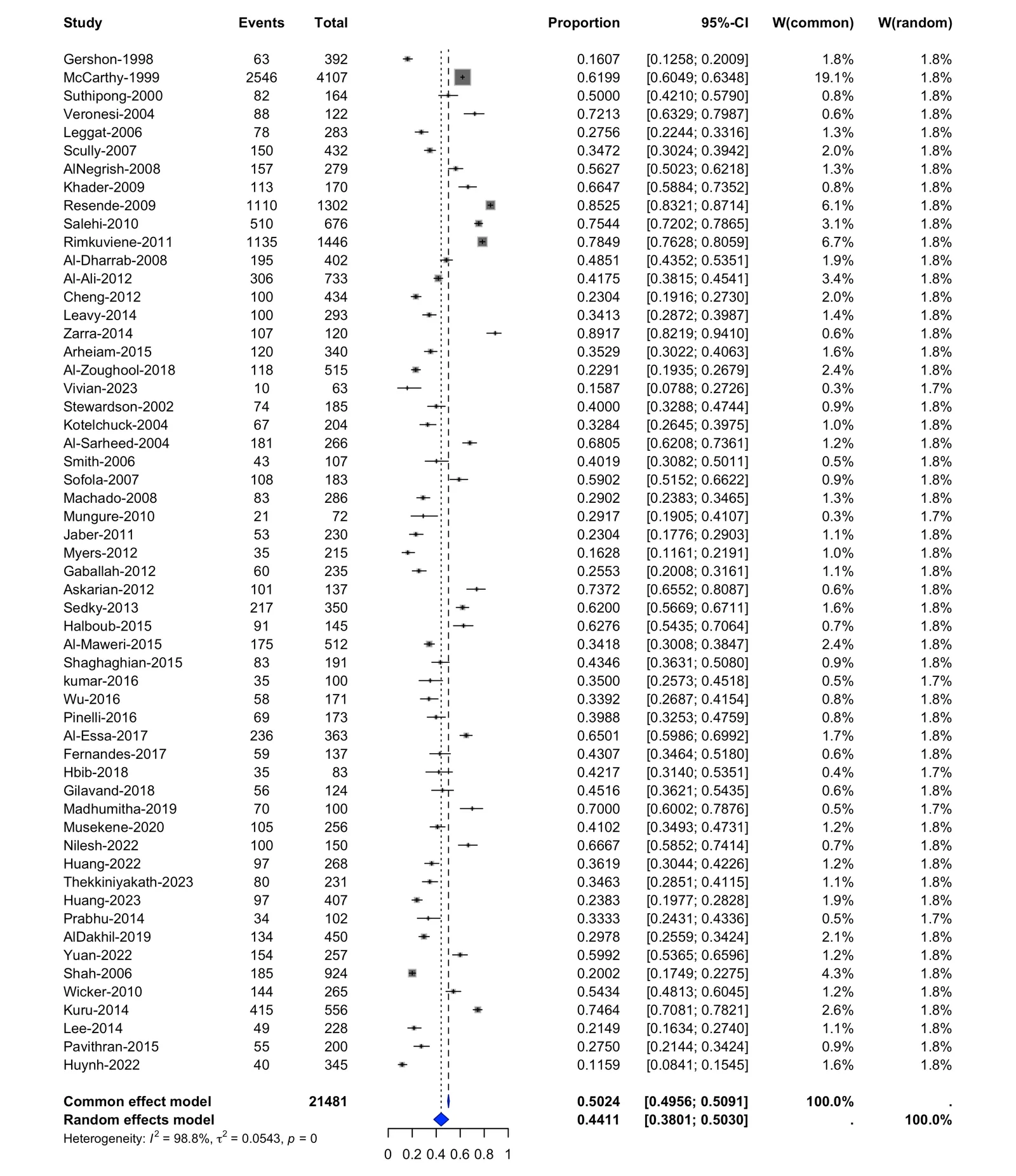
Overall pooled prevalence of NSI among dental health care workers. CI = confidence interval, NSI = needlestick and sharps injuries.

**Figure 3.**
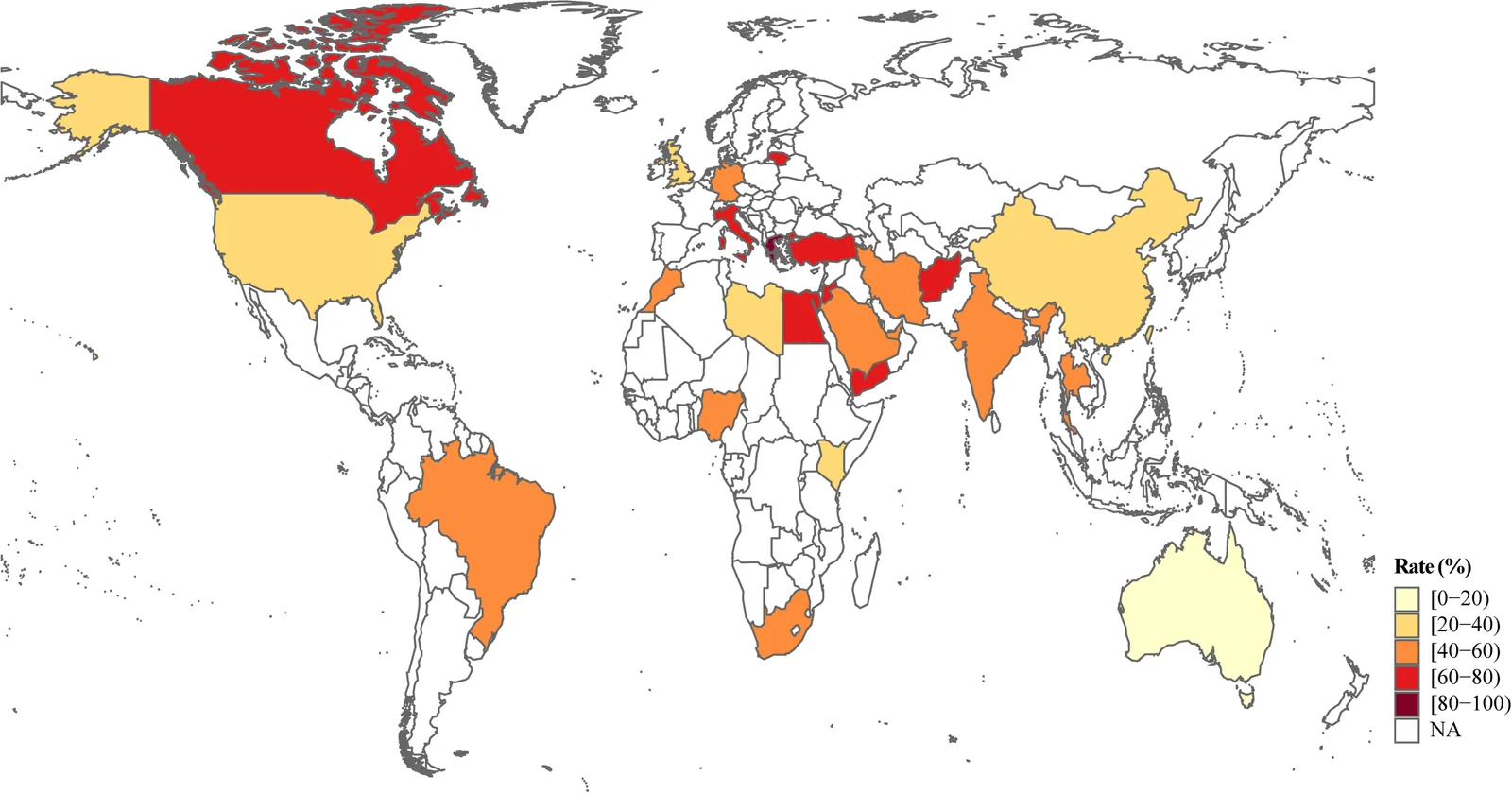
Map of NSI prevalence by country and region. NSI = needlestick and sharps injuries.

**Figure 4.**
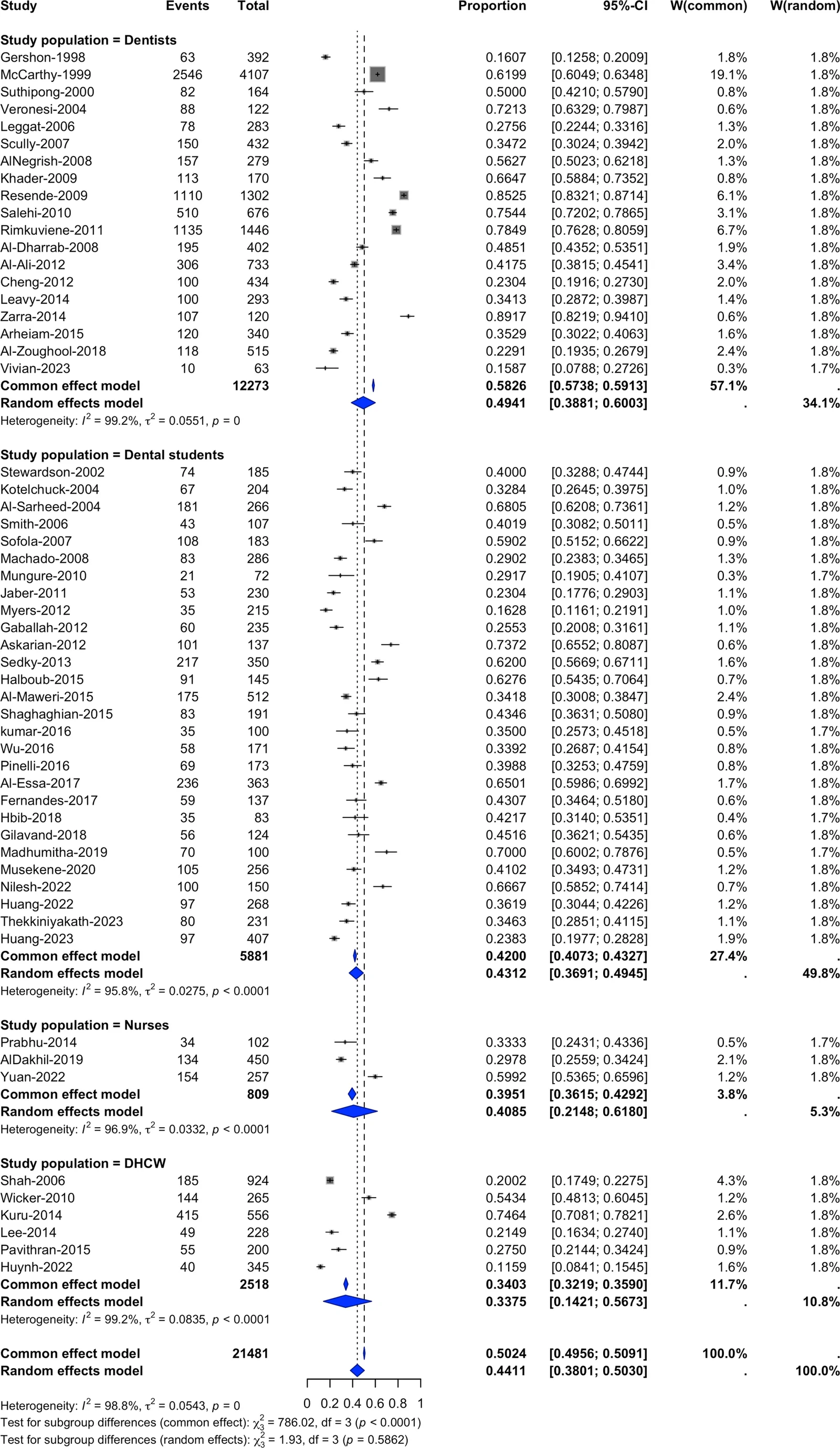
Subgroup analysis of the prevalence of NSI by occupational group (dentists, dental students, nurses, and other DHCWs). DHCW = dental health care worker, NSI = needlestick and sharps injuries.

### 3.4. Publication bias and sensitivity analysis

The funnel plot appeared relatively symmetrical, indicating no significant publication bias (Fig. S1, Supplemental Digital Content 1). Meanwhile, the sensitivity analysis using the leave-one-out approach and the trim and fill method confirmed the robustness of our findings (Fig. S2, Supplemental Digital Content 2).

## 4. Discussion

DHCWs are at elevated risk of NSI due to frequent use of dental burs, scalpels, suture needles, and local anesthetic syringes within confined clinical environments.^[10]^ A meta-analysis by Pereira et al reported that NSI prevalence among dentists ranged from 7.72% to 66.74%, with a pooled lifetime prevalence of 57.57%.^[11]^ DSS are especially vulnerable during early clinical training: Huang et al reported a prevalence of 44% among DSS, with consistently inadequate knowledge of NSI prevention. Notably, 66.1% of exposure events occurred when students worked without supervision.^[12]^ In recent years, dental institutions have increasingly employed dental assistants to handle instrument cleaning and transfer, many of whom have nonmedical backgrounds and limited protective awareness, contributing to an increasing incidence of sharps injuries.^[13]^ Dental nurses also face considerable risk owing to their direct handling of sharp instruments. Yuan et al investigated nurses at 3 dental hospitals in China and found that nearly 60% of nurses had experienced occupational exposure, highlighting the need for enhanced protective training.^[14]^ Inconsistent use of personal protective equipment and suboptimal infection-prevention awareness elevate the risk of occupational exposure; computer-vision-based monitoring of personal protective equipment adherence may help mitigate this gap.^[15,16]^ In addition to physical injury, NSI may cause psychological distress – including anxiety, fear, and insomnia – stemming from apprehension regarding BBPs such as HBV, HCV, and HIV, whereas most healthcare institutions currently lack dedicated psychological support services.^[17,18]^ DHCWs who acquire HIV/HBV/HCV may encounter practice restrictions, social stigma, and discrimination.^[19]^ Provision of psychological support and counseling to exposed workers is essential for mitigating anxiety and fear.^[20]^

Percutaneous injuries in dental settings occur most frequently among DHCWs with limited clinical experience. Protective training should encompass all DHCWs, with particular emphasis on high-risk groups including resident students and junior personnel.^[21,22]^ A 19-year retrospective review of risk factors for dental NSI at a Japanese university dental hospital reported that 58.4% of NSI occurred among practitioners with fewer than 5 years of clinical experience.^[23]^ A retrospective study of percutaneous injuries at polyclinic dental clinics in Singapore from 2014 to 2020 revealed that all dental personnel are at risk, with injuries concentrated among frontline clinical personnel; frontline dental workers with limited clinical experience were the predominant group affected.^[24]^ A retrospective review of percutaneous exposure incidents (PEIs) at an Australian dental school from 2009 to 2019 identified 618 reported PEIs, 79.9% of which occurred among students; local anesthesia injections were the most frequent injury scenario.^[25]^ Dental instruments are small and sharp; improper handling can readily result in percutaneous injury. Local anesthetic needles, burs, and endodontic files are the most frequently implicated devices; the most common puncture site is the finger pad of the nondominant hand. High-risk procedures include local anesthetic injection, endodontic treatment, and instrument handling, with 2-handed recapping contributing a substantial proportion of NSI. Clinically, local anesthesia administration, scaling, and waste disposal are the procedures associated with the highest NSI risk.^[11,23,26]^ Evidence indicates that needlestick injury incidence peaks toward the end of the workday and during breaks.^[27,28]^ Strengthening administrative supervision, optimizing staffing levels, and mitigating work-related fatigue among DHCWs represent effective protective measures against NSI.^[29]^ Innovative training modalities, including virtual reality (VR) simulation and scenario-based role-playing, have been demonstrated to enhance skill acquisition.^[30,31]^

Cultivating a safety culture, enhancing universal safety awareness, and establishing a nonpunitive reporting system are prerequisites for effective prevention and management.^[22,29]^ A review of 9775 NSI records across 252 hospitals in Poland found that 45.2% of NSI were underreported, highlighting that training programs should prioritize improving reporting rates.^[32]^ An evaluation of primary dental care practices in Scotland similarly identified deficiencies in reporting mechanisms and attitudes toward incident documentation.^[26]^ At the individual protection level, treating all patients as potential sources of BBPs and implementing standard precautions are essential.^[33]^ Wearing double gloves during high-risk procedures such as local anesthesia and oral surgery provides enhanced protection.^[34,35]^ However, practices prohibited by multiple national guidelines – such as 2-handed recapping and manual passing of sharps – remain prevalent, reflecting inadequate enforcement and an absence of safety culture.^[11,23,26]^ Engineering controls constitute the primary approach to NSI prevention, designed to minimize exposure risk at the source through safety-engineered syringes, puncture-resistant endodontic files, and anesthetic needle recapping devices. The US CDC lists safety-engineered devices as the primary engineering control and recommends the 1-handed scoop technique.^[36]^ The WHO recommended universal adoption of safety-engineered syringes by 2020; however, dental-specific safety devices remain constrained by cost and clinical acceptance.^[37]^ Huang et al applied the Joanna Briggs Institute PACES + GRiP framework and achieved an increase in safety syringe utilization from 16% to 95.5% and proper sharps disposal from 75% to 80%, suggesting that training may facilitate the adoption of engineering controls.^[38]^ Given the limited high-level evidence, controversy persists regarding immediate wound management: guidelines from the United States, New Zealand, and Australia recommend immediate irrigation of the wound with soap and running water and explicitly prohibit squeezing,^[20,39,40]^ whereas guidelines from China and the Dutch KNMT recommend gently expressing blood from proximal to distal before irrigation.^[41,42]^

## 5. Limitations

Several limitations of this study should be acknowledged. First, subgroup analyses stratified by healthcare setting, professional experience, and region were limited by incomplete reporting in the primary studies. Second, the observational design of the included studies constrains causal inference and limits evidence quality. Third, the considerable between-study heterogeneity (*I*^2^ = 98.77%) may reflect variations in practice environments, experience levels, and geographic regions and cannot be fully accounted for. Finally, the variable methodological quality of the included studies further undermines the robustness of our findings. Well-designed prospective cohort studies are required to validate these estimates.

## 6. Conclusion

Despite these limitations, the pooled NSI prevalence among DHCWs remains elevated. Most existing evidence is derived from general hospital settings, whereas dental practice entails unique instrument-related risks that warrant independent consideration. Priority measures for enhancing dental occupational safety include promoting needlestick injury prevention training programs, accelerating the development of dental-specific safety devices, strengthening post-exposure psychological support services, and establishing a comprehensive management system from prevention and reporting to post-exposure management and continuous improvement.

## Author contributions

**Conceptualization:** Xiaodan Zhu, Weili Ding, Mao Ye, Ling Zhang, Min Xing, Wenhao Qian.

**Data curation:** Xiaodan Zhu, Weili Ding, Mao Ye, Bei Zhang.

**Formal analysis:** Xiaodan Zhu.

**Funding acquisition:** Xiaodan Zhu.

**Investigation:** Xiaodan Zhu.

**Methodology:** Xiaodan Zhu, Weili Ding.

**Project administration:** Wenhao Qian

**Supervision:** Weili Ding, Mao Ye, Ling Zhang, Min Xing, Wenhao Qian.

**Validation:** Xiaodan Zhu, Ling Zhang, Bei Zhang.

**Visualization:** Xiaodan Zhu, Weili Ding.

**Writing – original draft:** Xiaodan Zhu, Weili Ding.

**Writing – review & editing:** Mao Ye, Ling Zhang, Min Xing, Wenhao Qian.




